# Kaempferol Induces G2/M Cell Cycle Arrest via Checkpoint Kinase 2 and Promotes Apoptosis via Death Receptors in Human Ovarian Carcinoma A2780/CP70 Cells

**DOI:** 10.3390/molecules23051095

**Published:** 2018-05-05

**Authors:** Ying Gao, Junfeng Yin, Gary O. Rankin, Yi Charlie Chen

**Affiliations:** 1 Tea Research Institute Chinese Academy of Agricultural Sciences, Ministry of Agriculture, Hangzhou 310008, China; yinggao@tricaas.com (Y.G.); yinjf@tricaas.com (J.Y.); 2 Department of Biomedical Sciences, Joan C. Edwards School of Medicine, Marshall University, Huntington, WV 25755, USA; rankin@marshall.edu; 3 College of Science, Technology and Mathematics, Alderson Broaddus University, Philippi, WV 26416, USA

**Keywords:** kaempferol, cell cycle arrest, Chk2, apoptosis, DR5, Fas

## Abstract

Kaempferol is a widely distributed dietary flavonoid. Epidemiological studies have demonstrated kaempferol consumption lowers the risk of ovarian cancer. Our previous research proved that kaempferol suppresses human ovarian cancer cells by inhibiting tumor angiogenesis. However, the effects of kaempferol on the cell cycle and extrinsic apoptosis of ovarian cancer cells have not yet been studied. In the present study, we demonstrated that kaempferol induced G2/M cell cycle arrest via the Chk2/Cdc25C/Cdc2 pathway and Chk2/p21/Cdc2 pathway in human ovarian cancer A2780/CP70 cells. Chk2 was not responsible for kaempferol-induced apoptosis and up-regulation of p53. Kaempferol stimulated extrinsic apoptosis via death receptors/FADD/Caspase-8 pathway. Our study suggested that Chk2 and death receptors played important roles in the anticancer activity of kaempferol in A2780/CP70 cells. These findings provide more evidence of the anti-ovarian cancer properties of kaempferol and suggest that kaempferol could be a potential candidate for ovarian cancer adjuvant therapy.

## 1. Introduction

**Figure 1:**
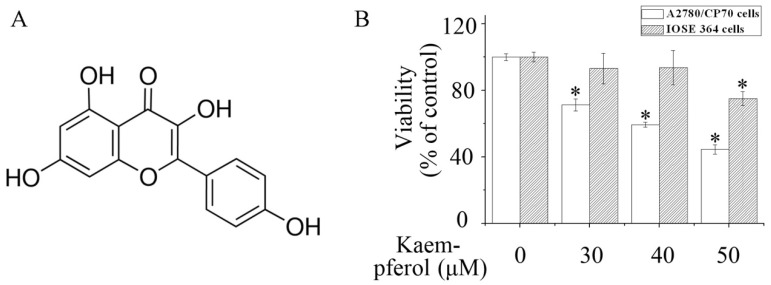
Kaempferol preferentially inhibited the viability of human ovarian carcinoma A2780/CP70 cells. (**A**) Chemical structure of kaempferol; (**B**) The effects of keampferol on the viability of A2780/CP70 cells and IOSE-364 cells. * P < 0.05 compared with the control group.

Kaempferol (Figure 1A) is a widely distributed natural flavonoid. It occurs not only in various fruits and vegetables (e.g., apples, berries, broccoli), but also in many traditional medical herbs (e.g., *Kaempferia rotunda* L., *Hedyotis diffusa* Willd, *Hypericum perforatum*). Numerous studies have demonstrated that kaempferol and some kinds of kaempferol glycosides exert a wide range of physiological activities, including antioxidant, anti-inflammatory, anti-microbial, anticancer, anti-diabetic, and anti-obesity activities [1].

Recently, an epidemiological study has shown that kaempferol consumption is associated with a linear decline in ovarian cancer risk [2]. Ovarian cancer is a gynecological cancer with poor prognosis. The estimated new ovarian cancer cases and deaths in the United States in 2018 are 22,240 and 14,070, respectively [3]. Most ovarian cancer patients die because of delayed diagnosis or recurrent disease [4]. Cytoreductive surgery with chemotherapy is the standard of care for ovarian cancer. However, the present treatment rarely works in patients with advanced-stage or recurrent ovarian cancer, and may cause severe systemic toxicity [5]. Therefore, it is essential to develop more efficient and safer cancer treatments. Flavonoids, a class of plant secondary metabolites, are regarded as prospective compounds for cancer prevention and anticancer therapy because of their high effectiveness and few side effects [6,7]. Checkpoint kinase 2 (Chk2) and death receptors have been reported to be the targets of flavonoids [8,9,10,11].

Chk2, a stable serine/threonine kinase expressed throughout the cell cycle, is a tumor suppressor which regulates multiple fundamental cellular functions [12]. Mutations and/or deletions of Chk2 have been linked to a wide range of cancers [12]. Chk2 can be phosphorylated at threonine 68 and activated in response to DNA damage [13]. Active Chk2 acts as a signal transducer and phosphorylates a variety of substrates, such as the Cdc25 phosphatases, p53 and E2F1, which are associated with the induction of the cell cycle arrest, the initiation of DNA repair, and the activation of apoptosis [14]. 

Death receptors are members of the tumor necrosis factor receptor superfamily characterized by a cytoplasmic region known as the “death domain” [15]. DR5 (also known as TRAILR2) and Fas (also known as CD95) belong to the death receptor family. The binding of death receptors with their corresponding ligands results in the transduction of apoptotic and/or survival signals. For DR5 and Fas, they only activate apoptotic pathways [16]. Up-regulation of death receptors is not only a common strategy shared by many chemotherapy drugs to induce apoptosis of cancer cells [17,18], but also is related to overcoming drug resistance of cancer cells [19,20]. 

Our previous research revealed that kaempferol induced human ovarian cancer cells through activating the p53 pathway [21] and decreasing angiogenesis through ERK-NFκB-cMyc-p21 pathway [22]. In this study, we investigated whether kaempferol could interrupt the cell cycle and trigger extrinsic apoptosis in human ovarian cancer A2780/CP70 cells. The possible underlying mechanisms were also explored.

## 2. Results

### 2.1. Kaempferol Inhibits the Viability of A2780/CP70 Cells

To assess cell viability, the CellTiter 96^®^ Aqueous One Solution Cell Proliferation Assay was performed. Kaempferol dose-dependently inhibited the viability of human ovarian cancer A2780/CP70 cells. When treated with 40 μM kaempferol for 48 h, the viability of A2780/CP70 cells was reduced to 59% (Figure 1B). Meanwhile, kaempferol elicited less cytotoxicity to human normal ovarian epithelial IOSE 364 cells (Figure 1B).

### 2.2. Kaempferol Induces G2/M Cell Cycle Arrest in A2780/CP70 Cells

**Figure 2:**
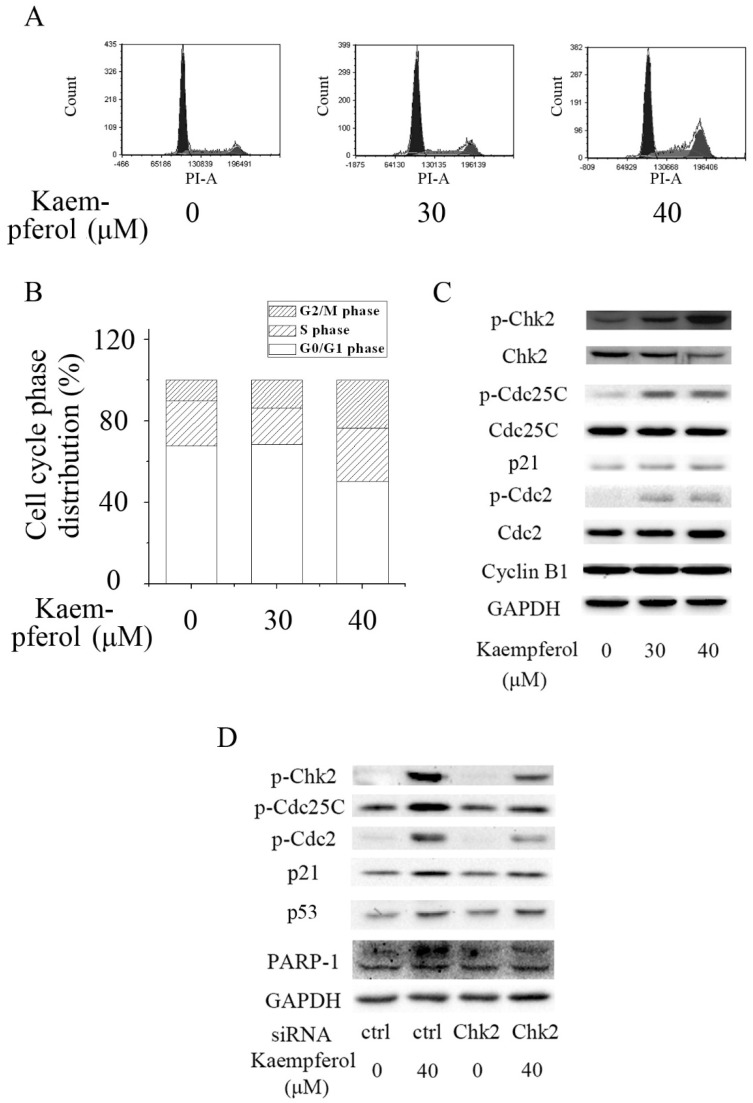
Kaempferol induced G2/M cell cycle arrest in A2780/CP70 cells via Chk2. (**A**,**B**) Flow cytometry assays revealed that kaempferol induced G2/M cell cycle arrest in A2780/CP70 cells; (**C**) Kaempferol increased the expression of p-Chk2, p-Cdc25C, p21 and p-Cdc2, but had no influence on the expression of Cyclin B1 in A2780/CP70 cells; (**D**) Knockdown of Chk2 attenuated kaempferol-induced up-regulation of p-Chk2, p-Cdc25C, p21 and p-Cdc2, but had no effect on the expression of p53 and cleavage of PARP-1 in A2780/CP70 cells. Glyceraldehyde-3-phosphate dehydrogenase (GAPDH) served as the loading control. Ctrl is short for control.

To measure the cell cycle distribution of A2780/CP70 cells after kaempferol treatment, cells were stained by PI and analyzed using flow cytometry. Cell cycle analysis revealed that kaempferol effectively induced an increased population of cells in the G2/M phase, suggesting kaempferol led to G2/M cell cycle arrest in A2780/CP70 cells (Figure 2A,B).

### 2.3. Kaempferol Induces Cell Cycle Arrest via Chk2

The cell cycle is driven by cyclin dependent kinases (CDKs) that associate with cyclin regulatory proteins at different points of the cell cycle. The activity of the Cyclin B1-Cdc2 complex is pivotal in the G2/M transition. Cdc2 is activated via dephosphorylating at Tyr15 and Thr14 during progression into mitosis. Cdc25C, which can be phosphorylated at Ser216 and inactivated by active Chk2, is a protein phosphatase responsible for activating Cdc2. P21, which can be induced by activation of Chk2 in a p53-independent manner [23], is another regulator of Cdc2 [24]. 

Western blot analysis demonstrated that the expression of p21 and the phosphorylation of Chk2, Cdc25C, and Cdc2 were dramatically enhanced after kaempferol treatment in A2780/CP70 cells (Figure 2C). The expression of Cyclin B1 was not influenced by kaempferol (Figure 2C). These results implied that kaempferol might induce G2/M cell cycle arrest by up-regulating the expression of p21 and inactivating G2/M phase-related proteins Cdc25C and Cdc2 through activating Chk2.

To explore the role of Chk2 in kaempferol-mediated G2/M cell cycle arrest in A2780/CP70 cells, Chk2 siRNA was transfected into A2780/CP70 cells to block the expression of Chk2. Knockdown of Chk2 partially abrogated kaempferol-induced up-regulation of p-Chk2, p-Cdc25C, p21 and p-Cdc2 (Figure 2D). The result indicated that kaempferol halted the cell cycle progression at G2/M phase via the Chk2/ Cdc25C/ Cdc2 pathway and Chk2/ p21/ Cdc2 pathway in A2780/CP70 cells.

### 2.4. Kaempferol Stimulates Apoptosis in A2780/CP70 Cells

Apoptosis is a form of programmed cell death [25]. The intrinsic (mitochondria-mediated) and extrinsic (receptor-mediated) pathways are two major apoptotic pathways. Caspase-8 and Caspase-9 are key initial caspases in the extrinsic apoptotic pathway and intrinsic apoptotic pathway, respectively. Activation of initial caspases leads to activation of effector caspases (e.g., Caspase-3 and Caspase-7) and cleavage of PARP-1.

**Figure 3:**
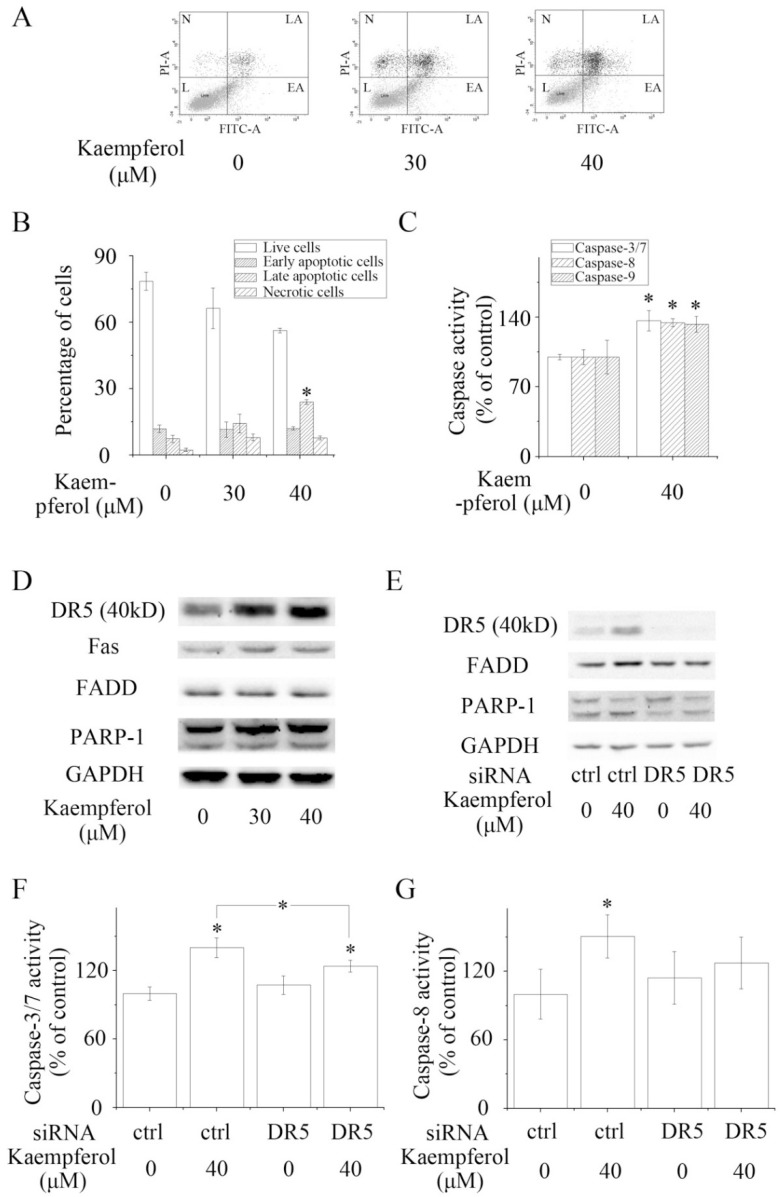
Kaempferol activated apoptosis in A2780/CP70 cells. (**A**,**B**) Flow Cytometry assays demonstrated that kaempferol induced apoptosis (mainly late apoptosis) in A2780/CP70 cells. N, LA, L and EA are short for necrotic cells, late apoptotic cells, living cells, and early apoptotic cells. (**C**) Kaempferol enhanced the activity of Caspase-3/7, -8 and -9 in A2780/CP70 cells. (**D**) Kaempferol promoted the expression of Fas, DR5, FADD, and cleaved PARP-1 in A2780/CP70 cells. (**E**–**G**) Knockdown of DR5 attenuated kaempferol-induced up-regulation of DR5, FADD and cleaved PARP-1, as well as the activation of Caspase-3/7 and Caspase-8. GAPDH served as the loading control. * P < 0.05 compared with control or between specific groups. Ctrl is short for control.

The result of flow cytometry assays revealed that kaempferol induced apoptosis in A2780/CP70 cells. The late apoptotic rate of A2780/CP70 cells was significantly increased to 23.95% when treated with 40 μM kaempferol for 48 h (Figure 3A,B). In accordance with the result of the flow cytometry assays, kaempferol significantly enhanced the cleavage of PARP-1 and the activity of Caspase-3/7, -8 and -9, respectively (Figure 3C). These results suggested that kaempferol induced apoptosis in A2780/CP70 cells via intrinsic and extrinsic pathways.

### 2.5. Kaempferol Initiates Apoptosis Not via Chk2 but via Death Receptors

It has been demonstrated that Chk2 can promote apoptosis in a p53-dependent manner. In the p53-dependent pathway, activated Chk2 mediates apoptosis through stabilizing and stimulating p53 [26,27]. Our previous work showed that kaempferol could induce intrinsic apoptosis via p53 [21]. To study whether Chk2 was involved in kaempferol-induced apoptosis, A2780/CP70 cells were transfected with Chk2 siRNA. Western blot analysis revealed that kaempferol-induced upregulation of p53 and cleavage of PARP-1 was not influenced by the knockdown of Chk2 (Figure 2D), suggesting that Chk2 was not involved in the kaempferol-induced apoptosis of A2780/CP70 cells and that kaempferol modulated the expression of p53 independently of Chk2.

As kaempferol enhanced the activity of Caspase-8, a key caspase in the extrinsic apoptotic pathway, we hypothesized that kaempferol might have an impact on the expression of death receptors and adaptor proteins. Western blot analysis showed that kaempferol increased the expression of two death receptors, DR5 and Fas, and one adaptor protein, FADD (Figure 3D). Blocking DR5 partially reversed kaempferol-induced up-regulation of the expression of FADD and activation of Caspase-3/7 and Caspase-8 (Figure 3E–G). Our data illustrated that kaempferol mediated apoptosis via death receptors/ FADD/ Caspase-8 pathway in A2780/CP70 cells.

### 2.6. Kaempferol Inhibits Ovarian Cancer OVCAR-3 Cells

**Figure 4:**
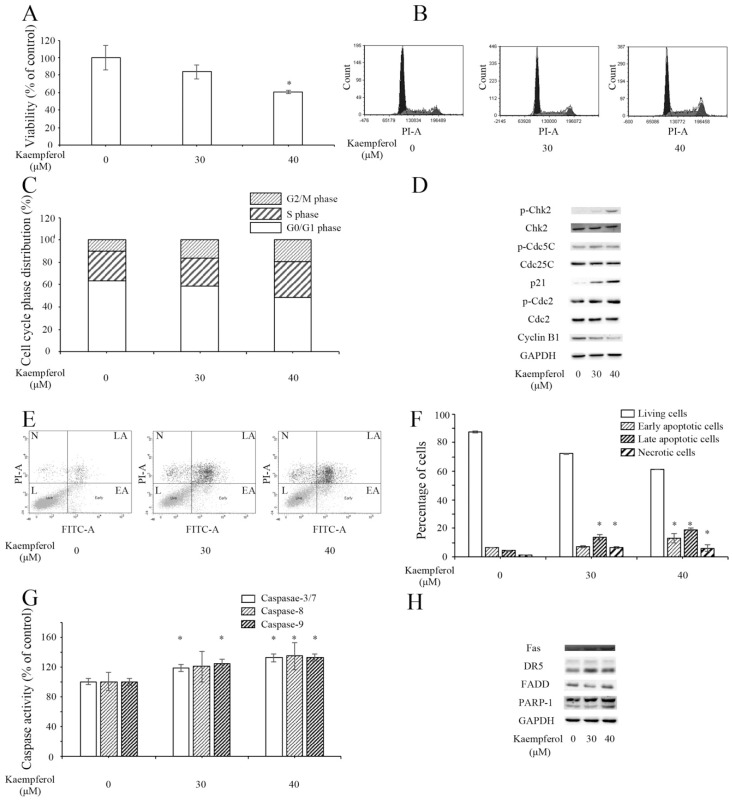
Anticancer activities of kaempferol on OVCAR-3 cells. (**A**) The effects of keampferol on the viability of OVCAR-3 cells. * P < 0.05 compared with the control group. (**B**,**C**) Flow cytometry assay revealed that kaempferol induced G2/M cell cycle arrest in OVCAR-3 cells. (**D**) Kaempferol influenced the expression of cell cycle-related proteins in OVCAR-3 cells. (**E**,**F**) Flow Cytometry assay presented that kaempferol induced apoptosis in OVCAR-3 cells. N, LA, L, and EA are short for necrotic cells, late apoptotic cells, living cells and early apoptotic cells. (**G**) Kaempferol enhanced the activity of Caspase-3/7, -8 and -9 in OVCAR-3 cells. (**H**) Kaempferol promoted the expression of Fas, DR5, and cleaved PARP-1 in OVCAR-3 cells.

To explore whether the anti-ovarian cancer activities of kaempferol were universal, we tested the inhibitory effects of kaempferol on another ovarian cancer cell line OVCAR-3. Results showed that the viability of OVCAR-3 cells was reduced to 60% under the treatment of 40 μM kaempferol for 48 h (Figure 4A). 

Kaempferol induced G2/M cell cycle arrest in a dose-dependent manner in OVCAR-3 cells (Figure 4B,C). Western blot assay revealed that kaempferol elevated the expression of p21 and activated the phosphorylation of Chk2, Cdc25C, and Cdc2 (Figure 4D). Meanwhile, it lowered the expression of Cyclin B1. 

Kaempferol triggered apoptosis in OVCAR-3 cells. The results of flow cytometry illustrated that the percentages of early apoptotic cells and late apoptotic cells were significantly increased in kaempferol-treated cells (Figure 4E,F), respectively. Kaempferol lifted the activities of caspases (Figure 4G). Kaempferol increased the expression of Fas, DR5, and cleaved PARP-1 (Figure 4H).

In general, kaempferol had a suppressive effect on OVCAR-3 cells. The main mechanisms were similar to the mechanisms of kaempferol on A2780/CP70 cells.

## 3. Discussion

Despite the improvement in diagnosis and treatment, cancer is still one of the most fatal diseases. Surgery, chemotherapy, and radiation therapy are three standard types of cancer treatment. Recently, hormonal therapy and targeted therapy have been used to treat various cancers. However, current cancer therapies have drawbacks. The traditional therapies might cause severe adverse side effects in patients. The newly developed therapies can only be applied to treat certain types of cancer. Thus, it’s necessary to find novel treatments or adjuvant therapies for treating cancer.

Flavonoids are regarded as promising anticancer agents. Some dietary flavonoids have cytotoxicity against various cancers, but have little or no side effects on normal cells. Kaempferol, a dietary flavonoid widely existing in vegetables and fruits, has anticancer activity. Our previous studies showed that kaempferol induced intrinsic apoptosis via activating p53 [21]. P53 is a major tumor suppressor whose function is critical for anticancer effects. However, loss and/or mutation of p53 is very common in human cancers [28]. Thus, we explored whether kaempferol had anticancer targets other than p53.

Chk2 emerges as an important signal transducer of cellular responses and a candidate tumor suppressor [29]. Activation of Chk2 leads to the phosphorylation of over 20 proteins. Cell cycle arrest, apoptosis, senescence, and DNA repair or tolerance of damage are induced by activation of Chk2 depending on the extent of damage, the cell type, and other factors [30].

Cdc25C and p21 are two down-stream proteins of Chk2. Both of them regulate the activity of Cdc2. Some flavonoids and isoflavones exert anticancer activities by targeting these proteins. Eupatilin, a naturally occurring flavonoid isolated from *Artemisia princeps*, has been reported to inhibit the growth of human endometrial cancer cells via activating the Chk2/Cdc25C/Cdc2 pathway [8]. Dihydromyricetin, a flavonoid commonly found in grapes, berries, vegetables, herbs, and other plants, induces G2/M phase cell cycle arrest of hepatocellular carcinoma cells through the Chk2/Cdc25C pathway [31]. Genistein, an isoflavone found in soybeans and other plants, caused G2/M cell cycle arrest in MCF-7 and MDA-MB-231 breast carcinoma cells via p21-mediated inhibition of Cdc2 [32]. In the present study, we observed that kaempferol induced G2/M cell cycle arrest via the Chk2/Cdc25C/Cdc2 pathway and Chk2/p21/Cdc2 pathway in A2780/CP70 cells. This result is in accordance with a previous study. In that case, kaempferol was proven to activate G2 cell cycle arrest through the Chk1/Chk2 pathway in human acute leukemia Jurkat T cells [33]. Our study and previous studies implied that Chk2 might be a universal target for the cell cycle-interrupting activity of flavonoids.

Death receptors refer to those members of the tumor necrosis factor receptor superfamily that contain a death domain, such as Fas, DR4, and DR5. Among them, DR5 is regarded as one of the most important anticancer targets. Up-regulation and/or activation of DR5 not only sensitizes cancer cells to apoptosis [34,35], but also collapses tumor blood vessels to reduce tumor growth [36]. Recently, DR5 was proven to act as a suppressor of human cancer cell invasion and metastasis [37]. Many studies have shown that DR5 inducers or agonists exert anticancer activities in cell models and animal models. Synergistic anticancer effects are also detected when DR5 inducers or agonists are combined with traditional chemotherapeutics (e.g., doxorubicin) [38]. Unlike other death receptors, the expression of DR5 is much lower in normal tissues than in cancerous tissues [36,39], implying that targeting DR5 might induce cancer-specific cell death and cause few adverse effects in normal cells. In the present study, kaempferol enhanced the expression of DR5, Fas, and FADD, the activity of Caspase-8 and cleavage of PARP-1. Knockdown of DR5 partially rescued kaemferol-induced activation of the extrinsic apoptotic pathway. Former studies illustrated that kaempferol increased the levels of membrane-bound FAS ligand, decreased uncleaved caspase-8 and intact Bid, and increased caspase-8 activity in HT-29 human colon cancer cells [40]. In human colon cancer SW480 cells, kaempferol markedly up-regulated tumor necrosis factor-related apoptosis-inducing ligand (TRAIL) receptors, DR5, and death receptor 4 (DR4) [41]. Kaempferol sensitized cancer cells to TRAIL-induced apoptosis via up-regulation of death receptors [41]. One of the mechanisms in which kaempferol modulated death receptors was by activating the reactive oxygen species-mediated p53/ ataxia telangiectasia mutated (ATM) signaling pathway [42]. Another study indicated that the JNK/ ERK-CHOP pathway was also involved [43]. Our results supplied additional evidence that death receptor signaling pathway played an important role in kaempferol-induced apoptosis.

## 4. Materials and Methods 

### 4.1. Cell Culture and Reagents

Human ovarian carcinoma cell lines A2780/CP70 and human immortalized ovarian surface epithelial cells (IOSE 364), were kind gifts from Dr. Bing-Hua Jiang at Thomas Jefferson University and Dr. Auersperg at the University of British Columbia, respectively. Cells were cultured in RPMI 1640 medium (Sigma, St. Louis, MO, USA) incorporating 10% fetal bovine serum (FBS) (Invitrogen, Grand Island, NY, USA). Cells were grown in a humidified incubator containing 5% CO_2_ at 37 °C.

Reagents: Kaempferol was purchased from Sigma. Dead Cell Apoptosis Kit with Annexin V Alexa Fluor^®^ 488 and propidium iodide (PI) was purchased from ThermoFisher Scientific (Waltham, MA, USA). Caspase-Glo^®^ 3/7 Assay Systems, Caspase-Glo^®^ 8 Assay Systems and Caspase-Glo^®^ 9 Assay Systems were purchased from Promega (Madison, WI, USA). Antibodies against p21 Waf1/Cip1 (p21), p-Cdc25C (Ser 216), Cdc25C, p-Cdc2 (Tyr 15), Cdc2, Cyclin B1, p53, death receptor 5 (DR5), Fas, and Fas-Associated protein with Death Domain (FADD) were purchased from Cell Signaling Technology, Inc. (Danvers, MA, USA). Antibodies against phosphor-checkpoint kinase 2 (Chk2) (Thr68), Chk2, poly [ADP-ribose] polymerase 1 (PARP-1), and glyceraldehyde-3-phosphate dehydrogenase (GAPDH) were purchased from Santa Cruz Biotechnology Inc. (Santa Cruz, CA, USA).

### 4.2. Assessment of Cell Viability In Vitro

Cells were seeded into 96-well plates at a density of 10^4^ cells per well in medium with 10% FBS. After overnight growth, cells were treated with kaempferol for 48 h. Cell viability was measured using the CellTiter 96^®^ Aqueous One Solution Cell Proliferation Assay (Promega, Madison, WI, USA), according to the manufacturer's instructions. Cell viability was expressed as a percentage compared to the control group.

### 4.3. Cell Cycle Analysis

Cells were treated with kaempferol for 48h. Then cells were harvested and fixed with cold 70% ethanol overnight. Cells were stained according to the manufacturer's instructions. Data acquisition and analysis were performed following flow cytometry with accompanying software (FACS Calibur, version 5.1, BD Bioscience, San Jose, CA, USA).

### 4.4. Western Blot

Cells were treated with kaempferol for 48 h in 60 mm dishes and then harvested. The Western blot assay was conducted according to a previously published method [44]. Protein bands were quantified with the NIH ImageJ software (NIH), normalized to the corresponding GAPDH band for analysis.

### 4.5. Transfection with Small Interfering RNA (siRNA)

Cells were seeded and incubated overnight. Then cells were transfected with control or target-specific siRNA using jetPRIME™ DNA and siRNA transfection reagent (VWR International, Radnor, PA, USA) according to the manufacturer's protocol. After 24 h, cells were exposed to cell culture medium containing kaempferol (0 or 40 μmol/ L) for another 48 h.

### 4.6. Apoptosis Analysis

Cells were treated with kaempferol for 48 h. Then cells were collected and stained with Annexin V Alexa Fluor^®^ 488 and propidium iodide (PI) according to the manufacturer's instructions. Data acquisition and analysis were performed following flow cytometry with accompanying software (FACS Calibur; BD Bioscience, San Jose, CA, USA).

### 4.7. Caspase Activity Assay

Cells were seeded into 96-well plates at a density of 10^4^ cells per well in medium with 10% FBS. After overnight growth, cells were treated with kaempferol for 48 h. Caspase-3/7, Caspase-8, and Caspase-9 activities were detected using Caspase-Glo 3/7, Caspase-Glo 8 or Caspase-Glo 9 Assay kit (Promega), respectively. Caspase activities were expressed as a percentage compared to the control group.

### 4.8. Statistical Analysis

All data were expressed as mean ± standard error of mean (SEM) from at least three independent experiments. The results were analyzed with SPSS Version 18.0 for Windows (SPSS, Chicago, IL, USA) using one-way analysis of variance (ANOVA) and a post hoc test (2-sided Dunnett’s test) to test both overall differences and specific differences between each treatment and control. P values < 0.05 were considered statistically significant.

## 5. Conclusions

**Figure 5:**
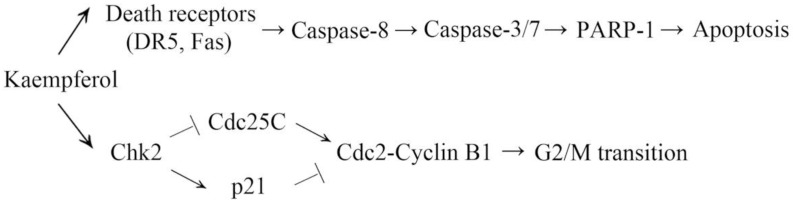
Proposed mechanism of inhibition of A2780/CP70 cells via Chk2 and death receptor signaling pathways.

Taken together, Chk2 and death receptors are pivotal anticancer targets of kaempferol (Figure 5). Kaempferol induced G2/M cell cycle arrest via the Chk2/Cdc25C/Cdc2 pathway and Chk2/p21/Cdc2 pathway. Chk2 was not involved in kaempferol-induced apoptosis. Kaempferol mediated intrinsic apoptosis via p53 in a Chk2-independent manner. It increased the expression of DR5 and Fas, resulting in the activation of extrinsic apoptosis. Since A2780/CP70 cells are cisplatin-resistant, our results suggested that kaempferol might help overcome cisplatin resistance in ovarian cancer. More studies are required to verify this hypothesis.
